# Novel animal model for Achilles tendinopathy: Controlled experimental study of serial injections of collagenase in rabbits

**DOI:** 10.1371/journal.pone.0192769

**Published:** 2018-02-13

**Authors:** Cesar de Cesar Netto, Alexandre Leme Godoy-Santos, Pedro Augusto Pontin, Renato Jose Mendonça Natalino, Cesar Augusto Martins Pereira, Francisco Diego de Oliveira Lima, Lucas Furtado da Fonseca, Jackson Rucker Staggers, Leonardo Muntada Cavinatto, Lew Charles Schon, Olavo Pires de Camargo, Túlio Diniz Fernandes

**Affiliations:** 1 Department of Orthopaedics and Traumatology, University of Sao Paulo (USP), School of Medicine, Sao Paulo, Sao Paulo, Brazil; 2 Department of Orthopaedics, Foot and Ankle Surgery, Medstar Union Memorial Hospital, Baltimore, Maryland, United States of America; 3 Department of Orthopaedics, Foot and Ankle Surgery, Hospital for Special Surgery (HSS), New York, New York, United States of America; 4 Department of Orthopaedics, University of Alabama at Birmingham (UAB), Birmingham, Alabama, United States of America; Mayo Clinic Minnesota, UNITED STATES

## Abstract

Our goal was to develop a novel technique for inducing Achilles tendinopathy in animal models which more accurately represents the progressive histological and biomechanical characteristic of chronic Achilles tendinopathy in humans. In this animal research study, forty-five rabbits were randomly assigned to three groups and given bilateral Achilles injections. Low dose (LD group) (n = 18) underwent a novel technique with three low-dose (0.1mg) injections of collagenase that were separated by two weeks, the high dose group (HD) (n = 18) underwent traditional single high-dose (0.3mg) injections, and the third group were controls (n = 9). Six rabbits were sacrificed from each experimental group (LD and HD) at 10, 12 and 16 weeks. Control animals were sacrificed after 16 weeks. Histological and biomechanical properties were then compared in all three groups. At 10 weeks, Bonar score and tendon cross sectional area was highest in HD group, with impaired biomechanical properties compared to LD group. At 12 weeks, Bonar score was higher in LD group, with similar biomechanical findings when compared to HD group. After 16 weeks, Bonar score was significantly increased for both LD group (11,8±2,28) and HD group (5,6±2,51), when compared to controls (2±0,76). LD group showed more pronounced histological and biomechanical findings, including cross sectional area of the tendon, Young’s modulus, yield stress and ultimate tensile strength. In conclusion, Achilles tendinopathy in animal models that were induced by serial injections of low-dose collagenase showed more pronounced histological and biomechanical findings after 16 weeks than traditional techniques, mimicking better the progressive and chronic characteristic of the tendinopathy in humans.

## Introduction

Achilles tendinopathy is one of the most common overuse injuries of the foot and ankle[1] and often results in chronic pain and functional impairment.[2,3] The disease is commonly diagnosed in physically active individuals, affecting 9% of recreational runners and up to 5% of professional athletes.[4] It also affects 5.6% of the sedentary population[5] and its chronic presentation is associated with advanced age.[6]

The pathogenesis of tendinopathy is multifactorial and is yet to be fully understood. [7]In general, we know that there is destruction of the tendinous micro-architecture, disorganization of collagen fibers, myxoid tissue degeneration, chondroid cellular metaplasia, deposition of intratendinous calcifications, and angiogenesis, resulting in decreased mechanical properties of the involved tendon.[8–10]

The continuum model of human tendinopathy proposed by Cook and Purdam in 2009, describe three consecutive and progressive stages of the disease depending on the frequency and intensity of the overload stimulus.[11] The initial reactive stage is characterized by non-inflammatory proliferative tissue reaction, with thickening of the tendon due to the up-regulation of large proteoglycans, with minimal collagen damage or separation. The second stage, or tendon disrepair, is characterized by a failed attempt of the tendon to heal, with greater tissue matrix breakdown, collagen separation, proliferation of abnormal tenocytes and some neovascularization. These two stages are considered to have some degree of reversibility. In the final stage of degenerative tendinopathy there is further disruption of collagen, widespread cell death and extensive neovascularization, leading to irreversible tendinopathic findings. [12]

The use of animal models are critical for helping us understand this complex disease because they allow for the study of each progressive stage of the disease in a controlled and reproducible environment, unlike human tissue, which is usually only obtained by biopsy or surgical resection during the more advanced stages of tendinopathy.[13] Furthermore, animal models are important in helping researchers develop better treatments for this debilitating disease.

The two most common techniques for inducing tendinopathy in animal models include mechanical overloading and chemical induction using collagenase, corticosteroids, prostaglandins, and cytokines.[14] The use of collagenase demonstrates particularly promising results in the chemical induction of Achilles tendinopathy, as shown in several studies. [15–20] This model is objective, easily reproducible, and requires minimal resources. Studies have shown that collagenase induced tendinopathy results in destruction and disorganization of collagen bundles, as well as cellular, molecular, and biomechanical alterations, resembling the most important features of human tendinopathy.[13,14,21,22] However, a major weakness of this model is that the current techniques with a single collagenase injection produce intense tendon alterations acutely, which does not accurately replicate the progressive and chronic nature of tendinopathy in humans.

To date, no protocols exist regarding optimal dosage, concentration, and frequency of injected collagenase to replicate chronic tendinopathy in humans. In previous studies, collagenase was empirically injected in a single dose of 0.3 to 0.5 milligrams.[22–29] A study by Orfei et al. was the first to compare histological findings after single 0.1mg or 0.3mg doses of collagenase.[19] To the best of our knowledge, there have been no studies evaluating the role of serial injections of collagenase in animal models of induced tendinopathy. We hypothesize that serial application of lower doses of collagenase, when compared to a single higher dose injection, would represent a repeated chemical overload to the tendon and could result in a more progressive and persistent tendinous alteration, replicating better the continuum model and the chronic nature of tendinopathy in humans.

## Material and methods

Institutional review board approval was obtained prior to this controlled experimental study. This animal study was approved by the University of Sao Paulo Medical School Ethics Committee [Comissão de Ética no Uso de Animais (CEUA) do Comitê de Ética em Pesquisa da Faculdade de Medicina da Universidade de São Paulo (CEP-FMUSP)], under the protocol number 148/12.

The number of animals for each group was calculated according to the Mead’s resource equation (E = N-T, 10<E<20), in a manner similar to Perucca et al.[19] Forty-five female New Zealand rabbits (*Oryctolagus cuniculus*) were used in the study (age 17–21 weeks, weight 3000-4000g). Exclusion criteria included: death during anesthetic procedure, cutaneous loss in the intervention area, autophagy and mutilation, deep postoperative infection, urinary tract infection characterized by gross hematuria, and weight loss greater than 20% during the follow-up period. All animals were randomly separated into three groups and given bilateral intratendinous injections of the Achilles tendon as described:

- Serial Low-Dose Injections (LD Group) - 18 animals given three 0.1mg injections of bacterial collagenase type A1 (Sigma-Aldrich, Saint Louis, Missouri, United States) (10 μL of solution containing 10 mg of collagenase for each 1 mL of 0.9% saline solution), separated by 14-day intervals.
- Single High-Dose Injection (HD Group) - 18 animals given a single 0.3 mg injection of collagenase type A1 (30 μL of solution containing 10 mg of collagenase for each 1 milliliter of 0.9% saline solution).
- Control Group (C Group)—nine animals given three injections of 10 μL of 0.9% saline solution, separated by 14 days intervals.

Animals were first anesthetized using intramuscular ketamine (40 mg/kg) and midazolam (2 mg/kg), which allowed trichotomy of the calcaneal region bilaterally. Fluid maintenance was performed via the saphenous vein with 10mL/kg/h of 0.9% saline solution (NS). Propofol (5mg/kg) was then administered intravenously, allowing orotracheal intubation and sedation with isoflurane diluted in 100% oxygen. Five minutes before the start of the surgical procedure the animals received intramuscular tramadol hydrochloride (5 mg/kg). Gentamicin was administered subcutaneously (4 mg/kg) immediately prior to the surgical procedure and once daily for the following five days.

**Fig 1:**
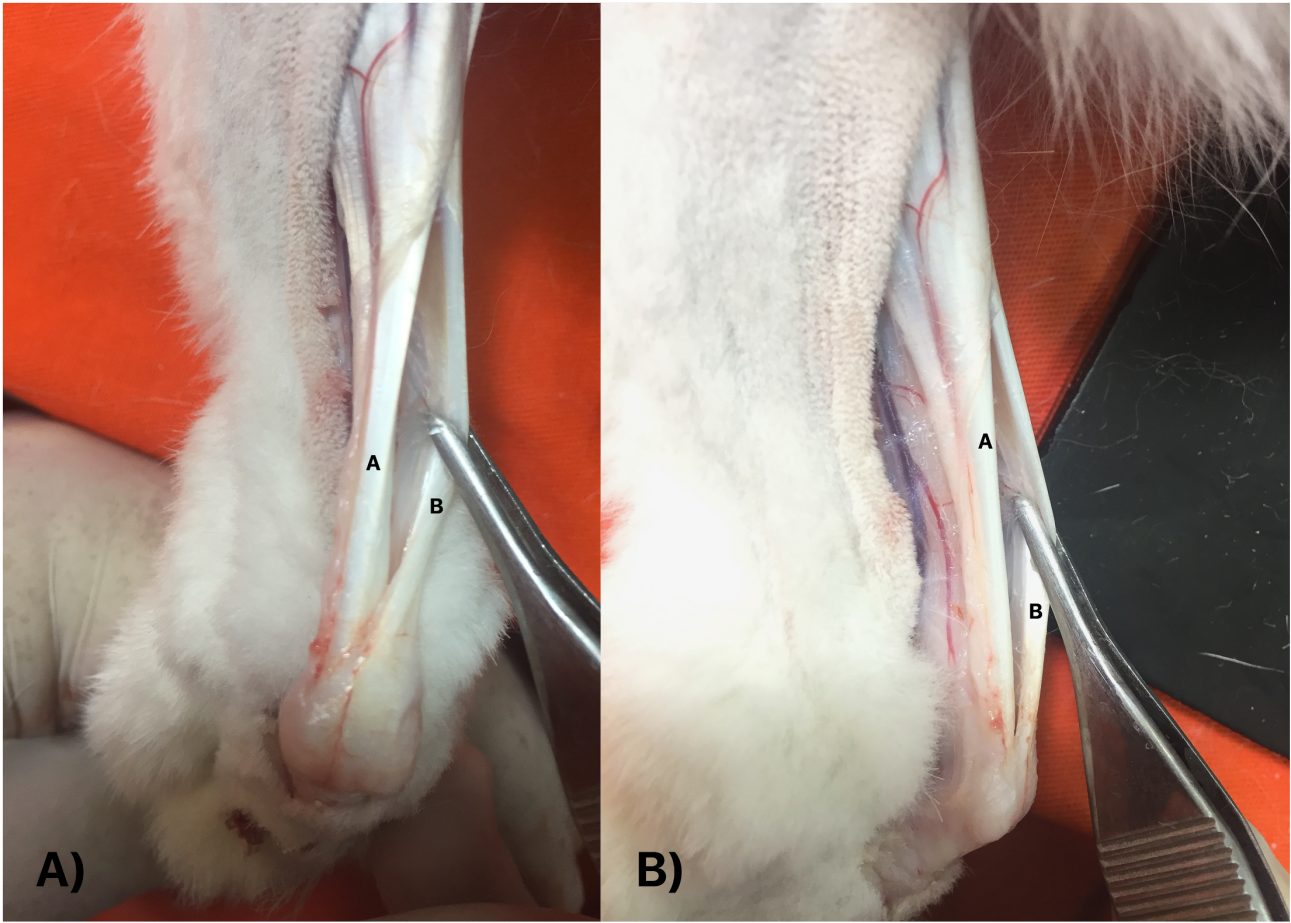
**Anatomic dissection of the Achilles (A) and plantaris tendons in rabbits (B).** (A) Posterior view. (B) Lateral view. The plantaris tendon is located in a superficial and dorsal position in relation to the Achilles tendon.

**Fig 2:**
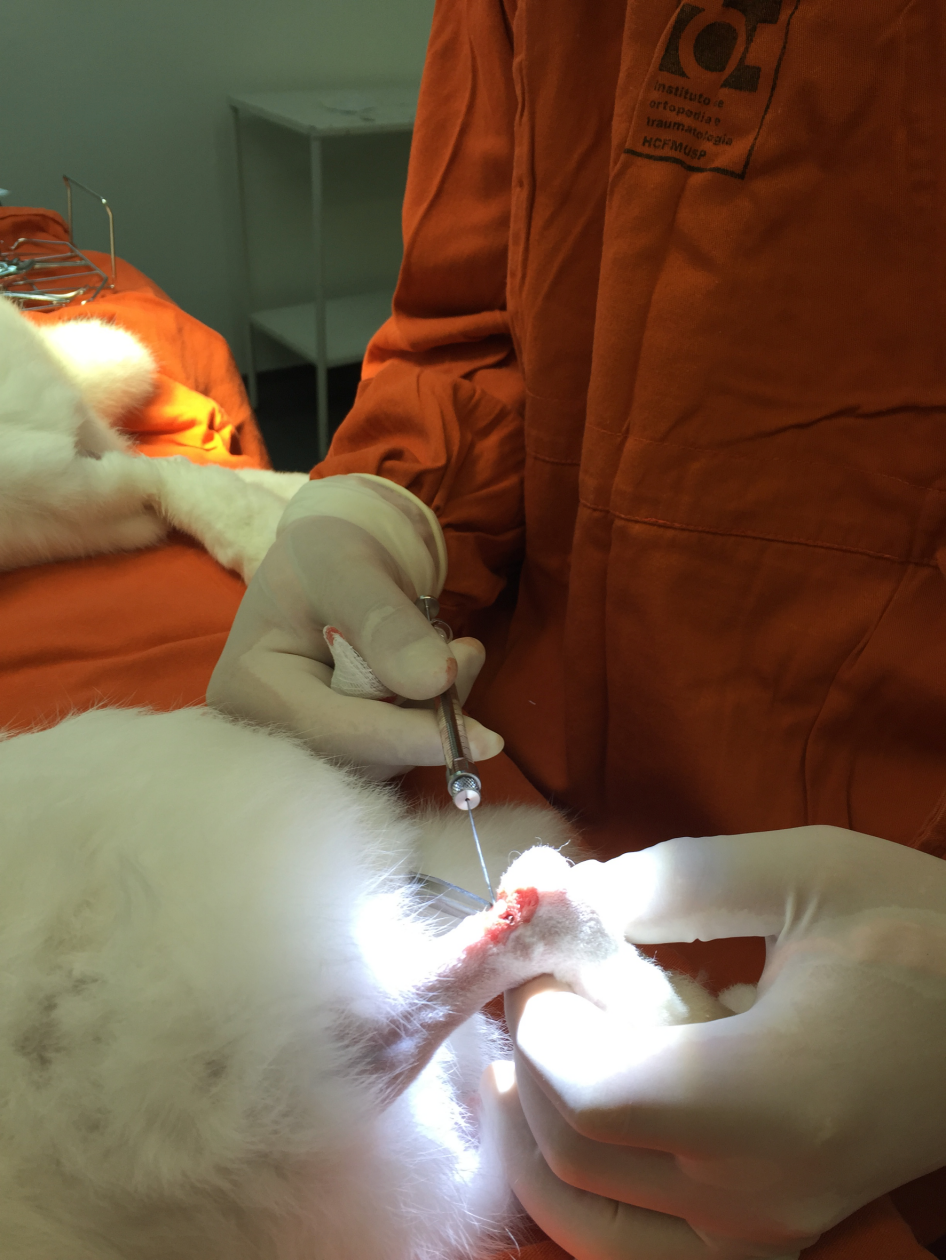
Tendon injection procedure. Injection performed under direct visualization, in the central region of the substance of the Achilles tendon, using 27 gauge needle coupled in a 100 microliters syringe.

Using sterile technique, the animal was positioned in ventral decubitus and a 1cm longitudinal incision was performed through a lateral approach to expose the Achilles and plantaris tendons.[23,30] The plantaris tendon represent a well-developed landmark structure in rabbits, and is located in a superficial and dorsal position in relation to the Achilles tendon (Fig 1).[31] After retraction of the plantaris muscle tendon, intratendinous injection of collagenase (LD and HD Groups) or saline solution (C Group) was performed with a 27G needle in the central region of Achilles tendon, 1.5cm proximal to its insertion at the calcaneal tuberosity (Fig 2). After the procedure, the surgical wound was closed with 5.0 nylon sutures, followed by sterile dressing. This procedure was then performed on the contralateral limb.

Following the procedure, the rabbits recovered in a climate controlled chamber (25°C to 28°C) for 30 minutes. Animals received analgesia with subcutaneous tramadol hydrochloride (3 mg/kg) every eight hours for the first 72 hours postoperatively. They were then kept in individual climate controlled cages (50 x 50cm, 20°C to 24°C) with appropriate nutrition and hydration, cycling 12h light/12h darkness. The rabbits’ general condition and operative wounds were monitored with daily veterinary evaluations. The body weight of each animal was measured weekly.

**Fig 3:**
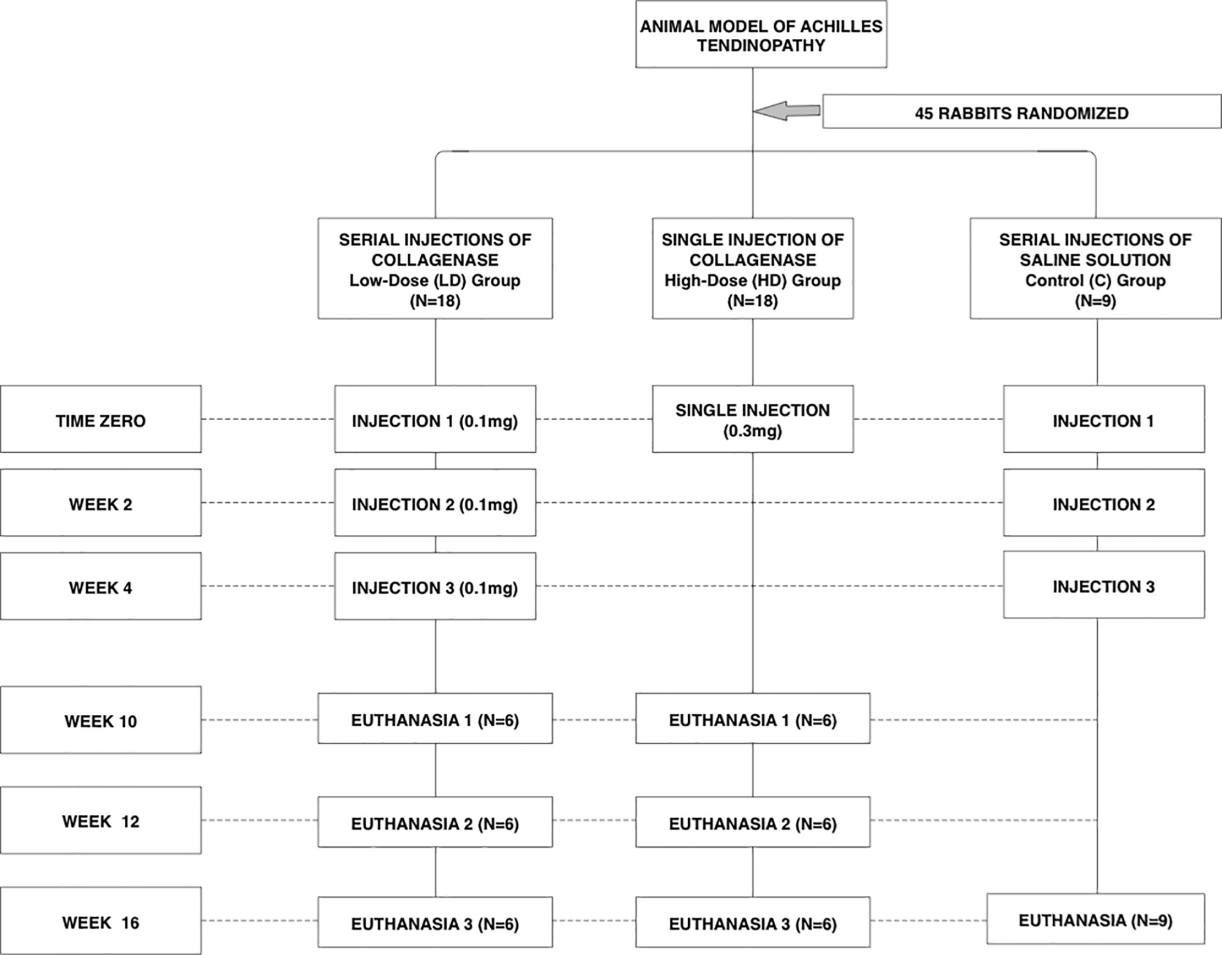
Distribution of the study groups. Flowchart for groups distributions, timing of injections and euthanasias.

Animals in the experimental LD and HD Groups were euthanized at three different time points (10, 12 weeks, and 16 weeks). The entire control group (C Group) was euthanized at 16 weeks (Fig 3).[22,32] All animals were euthanized according to the legislation in place. Sedation was obtained by intramuscular ketamine (40 mg/kg) and midazolam (2 mg/kg), followed by intravenous propofol (5 mg/kg). Animals were then euthanized with intravenous propofol (10 mg/kg), and 19.1% potassium chloride (10 mL).

The left and right pelvic limbs were dissected immediately after euthanasia for histological and biomechanical analysis, respectively. The right limbs were dissected to isolate the gastrocnemius and soleus muscles in continuity with the Achilles tendons and their insertion to the calcaneus. The plantaris muscle and tendon were resected and discarded. The length of the specimen were standardized at 55mm. Specimens were packed in gauze pads moistened with NS and stored at -18°C until submitted to biomechanical tests. The left limbs were dissected to isolate the entire length of the Achilles tendon. The macroscopic site with the most tendinopathy was identified and marked appropriately.

### Histological analysis

The tendon specimens to be used for histological analysis (left limbs) were fixed with 10% formaldehyde, diaphanized in xylol, and imbedded in paraffin. Longitudinal histological sections (5 microns) were made with a microtome (Leica HM 355S). Cuts were fixed on glass slides for subsequent staining and histological examination. Sections were stained by hematoxylin-eosin (H&E), alcian blue, safranin, and picrosirius techniques. The histological evaluation was performed blindly by two experienced musculoskeletal pathologist using a microscope (Zeiss Axioskop 2 Plus). Tendinopathic tissue changes were evaluated by histological criteria according to the *Bonar* score,[33,34] following the standardized recommendations of Fearon et al.[35]

**Fig 4:**
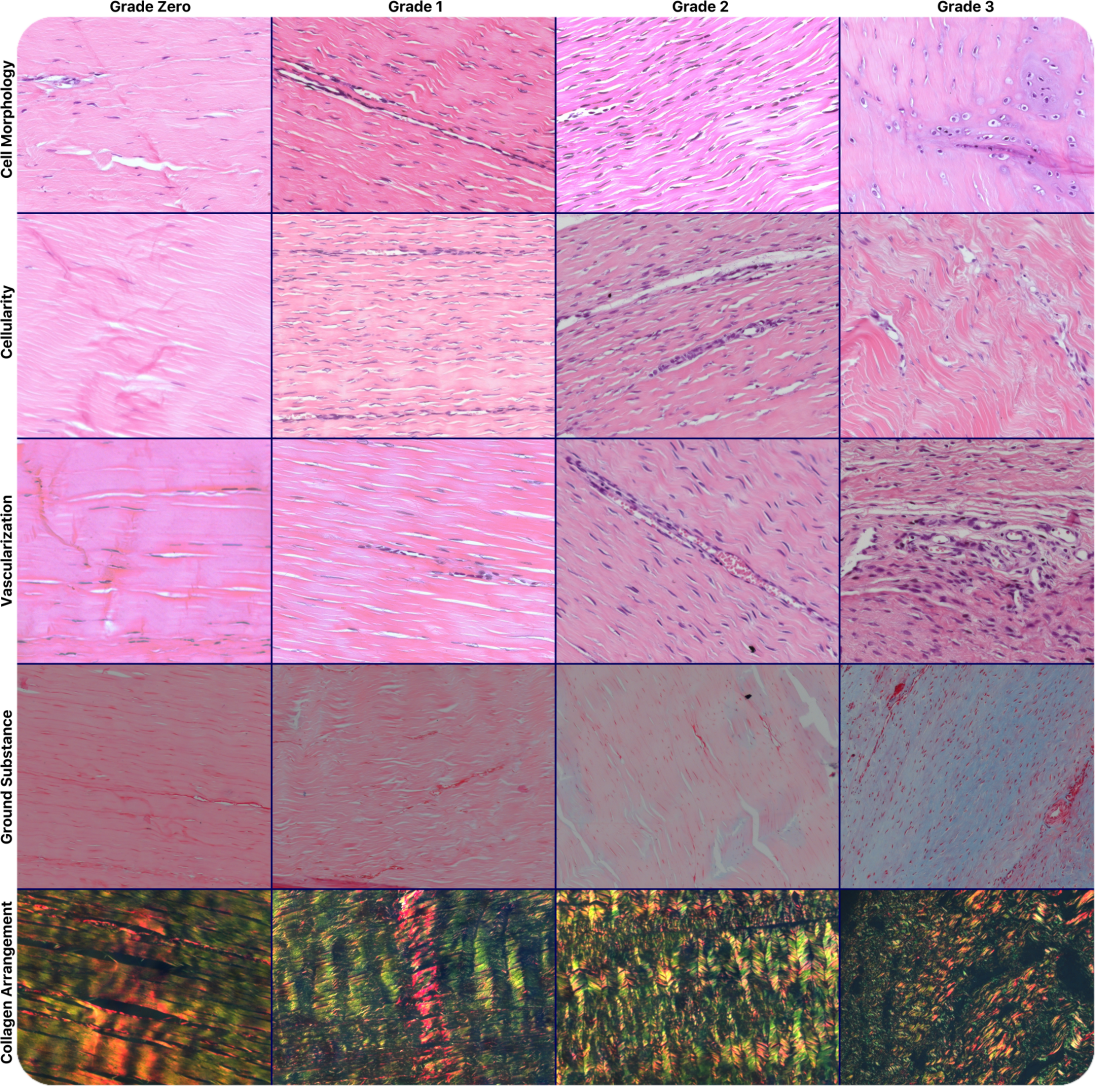
Histological *Bonar* score grading system. Five different characteristics evaluated, each one graded from zero to three: Line 1: cell morphology (200x magnification, H&E and safranin); Line 2: cellularity (100x magnification, H&E); Line 3: vascularization (400x magnification, H&E); Line 4: accumulation of ground substance (100x magnification, Alcian blue); Line 5: collagen fibers arrangement (polarized light, 100x magnification, picrosirius).

After the initial analysis of the entire tendon with 100x magnification, the region with the greatest alteration in cell morphology was identified and evaluated for the following five characteristics: collagen fiber arrangement (one field of view, polarized light, 100x magnification, picrosirius); cell morphology (four fields of view, 200x magnification, H&E and safranin); cellularity (one field of view, 100x magnification, H&E); vascularization (eight fields of view, 400x magnification, H&E); and accumulation of ground substance (one field of view, 100x magnification, Alcian blue). Each characteristic was graded from 0 (normal tendon tissue) to 3 (advanced changes) (Fig 4). The total Bonar score for each specimen was calculated from the sum of these five characteristic grades, ranging from 0–15, where a score of 0 represented a normal tendon, and a score of 15 represented a tendon with maximum pathological tendinopathic changes.

### Biomechanical analysis

**Fig 5:**
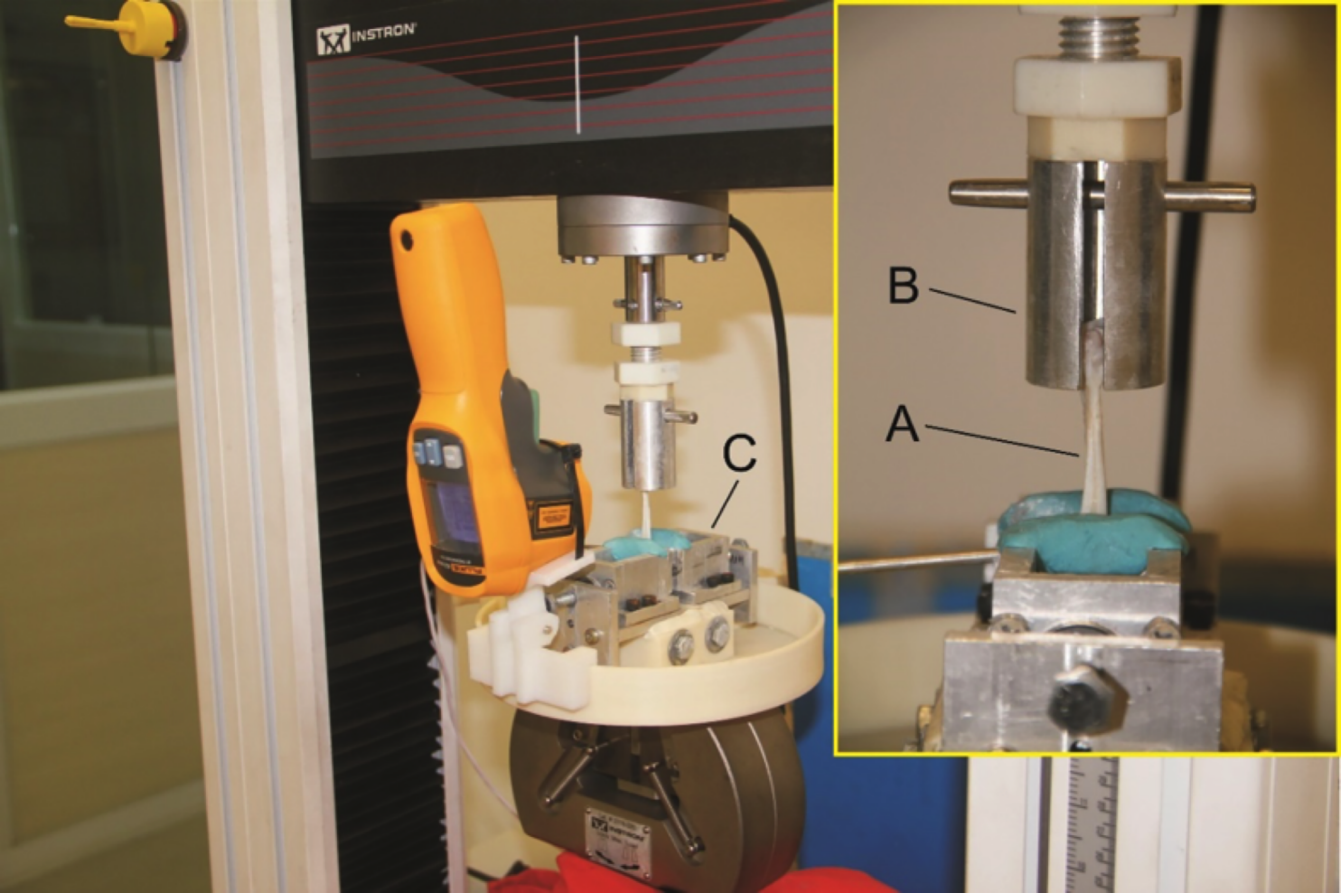
Biomechanical analysis. Specimen (A) attached to the test machine by a cylindrical (B) and a cryogenic clamp (C).

Biomechanical tests and data analysis was performed by blindly by a Ph.D. engineer. The specimens were thawed in NS (23°C), the Achilles tendon was measured with a caliper (Mitutoyo®)(± 0.01 mm) in three distinct regions: 35 mm proximal to the insertion, centrally, and at the muscle-tendon junction. The cross sectional area (mm^2^) was calculated from the mean value of these three measurements. Each specimen was then subjected to cyclic load preconditioning followed by destructive testing using an electromechanical test machine (Instron® model 3369) (± 0.5% accuracy) and controlled with appropriate software (Bluehill®). With the specimen properly positioned and fixed to the cryogenic clamps (Fig 5), a 5 N preload was applied for 30 seconds, followed immediately by biomechanical testing. To ensure that only the muscular end of the specimen froze, two temperature gauges were used to identify the ideal moment for the beginning of the mechanical test. Cyclical preconditioning of the specimen was performed totaling 50 repetitions (1 cycle/s) with loads ranging from 5N to 50N. Next, the destructive test was started once the temperature reached -5°C in the muscular region of the specimen and 10°C in the tendon substance.[17,36] The test was run at a speed of 1 mm/s and was terminated with complete rupture of the specimen occurred. The biomechanical parameters evaluated were stiffness (N/mm), *Young’s* modulus (MPa), yield stress (MPa) and yield tension (N), ultimate stress (MPa) and ultimate tension (N).

### Statistical analysis

Descriptive statistics were presented according to mean values and standard deviations. Data was initially evaluated by the *Kolmogorov-Smirnov* normality test and *Levene* homogeneity test. For the comparison between the groups, and within the groups at different follow-up time points, a two-way ANOVA test was performed. Interobserver reliability for histological Bonar score readings was assessed using Intraclass Correlation Coefficient (ICC), with consideration of the amount by which bias and interaction factors can reduce the ICC. Correlations of 0.81 to 0.99 were considered almost perfect. P-values of <0.05 were considered significant.

## Results

There were a total of seven losses (15.5%), including: two animals in LD group (11.1%), four in HD group (22.2%) and one in the C group (11.1%). Two deaths were due to anesthetic complications, two were sacrificed secondary to deep postoperative infection, one was sacrificed secondary to a fracture of the left hind paw inside the cage, one was secondary to an urinary infection, and there was one sudden death of unknown cause.

### Histological results

**Fig 6:**
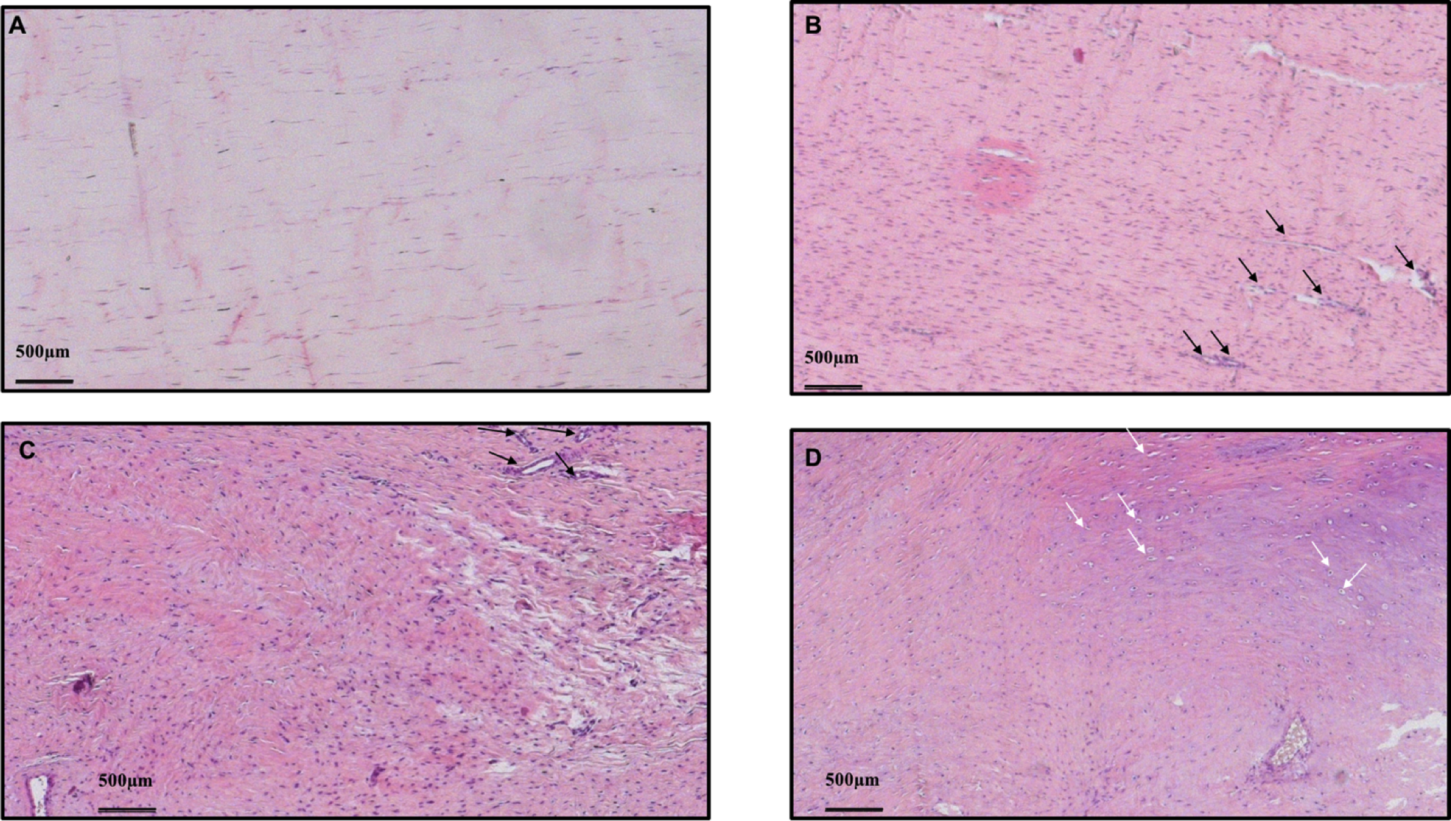
Histological results after 16 weeks. (A) Hematoxylin-Eosin, 40x. Control Group: cell morphology slightly altered, well delimited collagen bundles, minimal increase in cellularity, no changes in vascularization or ground substance. **(B)** HD Group: increased cellularity, altered cellular morphology with greater roundness of the cellular nuclei, loss of the wavy pattern of collagen bundles and areas of neovascularization (black arrows). (C) LD Group: Areas of increased, decreased and acellularity, destruction of collagen bundle architecture, increased number vascular clusters (black arrows) loss of collagen bundle architecture (D) LD Group: diffuse increase of the ground substance, altered cellular morphology, rounding of cell nuclei with halo formation, compatible with chondroid metaplasia (white arrows).

A summary of the histological results are shown in Table 1. The intraclass correlation coefficient (ICC) for interobserver reliability between the two independent and blinded readers was almost perfect (0.975). Thirty-eight rabbits (84.4%) were submitted to the histological study. Group, time-point and the interaction between them were found significantly influence the Bonar scores (p<0.0001) (Table 2). More specifically, in the LD group, Bonar scores after 10 weeks were significantly higher than after 12 weeks, with a mean difference 3.6 points (CI 95%; 1.49–5.71), and the values after 16 weeks were higher than after 12 weeks, with a mean difference of 1.69 points (CI 95%; 1.69–5.91). There was no difference when comparing findings after 10 and 16 weeks. Within the HD group, the Bonar score values after 10 weeks were significantly higher than the values after 12 and 16 weeks, with mean differences of respectively 8.3 (CI 95%; 5.97–10.63) and 7.2 points (CI 95%; 5.00–9.40) There was no significant difference in the comparison of specimens euthanized with 12 and 16 weeks. When comparing LD and HD groups at the 10-week mark, both groups had similar histological findings. At 12 weeks, LD group values significantly exceeded the HD group by 3.5 points (CI 95%; 0.32–4.68). At 16 weeks, the Bonar scores were significantly higher in LD group, when compared to both HD and control groups, with mean differences of respectively 6.20 (CI 95%; 4.00–8.40) and 9.80 points (CI 95%; 7.76–9.80) (Fig 6). Furthermore, the Bonar score in HD group was significantly higher than the control group, with a mean difference of 3.60 points (CI 95%; 1.56–5.64).

**Table 1:** Histological Bonar score: Mean values, standard deviation, Minimum, Median and Maximum values by Group at different euthanasia time-points.

Group	Time-Point	n	Mean	Std Dev	Minimum	Median	Maximum
**Control**	**16 weeks**	8	2	0.82	1	2	3
**High-Dose**	**10 weeks**	5	12.8	0.84	12	13	14
	**12 weeks**	4	4.5	1.91	2	5	6
	**16 weeks**	5	5.6	2.51	2	5	8
**Low-Dose**	**10 weeks**	5	11.6	0.55	11	12	12
	**12 weeks**	6	8	2.1	6	7.5	11
	**16 weeks**	5	11.8	2.28	8	12	14

n: number of animals

std dev: standard deviation

**Table 2:** Two-Way ANOVA analysis for histological Bonar score. Degrees of Freedom, Sum of Squares, Mean Square, F-Value and P-values. Significant influences observed for all variables: Group, Time-point (Weeks) and interaction between Group and Time-point.

Source		DF	Sum of Squares	Mean Square	F Value	p-value	
**Model**		6	580.676	96.779	33.37	< .0001	
	**Group**	2	336.524	168.262	58.02	< .0001	*
	**Week**	2	174.080	87.040	30.01	< .0001	*
	Group*Week	2	70.072	35.036	12.08	< .0001	*
**Error**		30	87.000	2.900			
**Total**		36	667.676				

DF: degrees of freedom

* p-value <0.05

### Biomechanical results

Thirty-four rabbits (75.6%) were submitted to the biomechanical study. This is due to the previously mentioned animal losses (n = 7) in addition to 4 more exclusions due to inadequate tissue samples for testing, including: 2 from LD group (12 weeks), 1 from HD group (16 weeks), and 1 from the control group.

During the destructive mechanical test, the site of the rupture in the specimen was recorded. In 28 (82.3%), rupture occurred at the insertion of the Achilles tendon in the calcaneal tuberosity. In the other 6 specimens, the rupture occurred near the cryogenic clamp, in the region of the muscle-tendon junction (17.7%).

A summary of the biomechanical results in each study group at different follow-up time points were presented below for 10 (Table 3), 12, (Table 4) and 16 weeks (Table 5). The type of injection (LD, HD or control) was found to significantly influence all biomechanical parameters evaluated, with the exception of yield tension. The time variable, for the comparison within each group at different time points, was found to be significant only for the cross-sectional area evaluation of the tendons. The interaction between group and time point was found to significantly influence Young’s modulus, yield stress, ultimate tension, ultimate stress and the cross-sectional area of the specimens.

**Table 3:** Biomechanical parameters at 10 weeks: Mean value and standard deviation for specimens in the Low-Dose (LD) and High-Dose (HD) Groups.

Biomechanical Parameters at 10 weeks (Mean Value ± SD)	Low-Dose Group (n = 5)	High-Dose Group (n = 5)
Cross Sectional Area (mm^2^)	16.59 ± 5.40	27.17 ± 7.87
Stiffness (N/mm)	84.26 ± 33.11	43.43 ± 13.61
*Young’s* Modulus (MPa)	219.63 ± 135.50	74.99 ± 43.97
Yield Tension (N)	271.10 ± 87.39	117.56 ± 42.36
Yield Stress (MPa)	17.91 ± 9.92	4.72 ± 2.14
Ultimate Tension (N)	389.14 ± 105.67	168.17 ± 59.59
Ultimate Stress (MPa)	25.08 ± 10.58	7.13 ± 4.16

SD: standard deviation

n: number of animals

mm: millimeters

N: Newtons

MPa: Megapascal

**Table 4:** Biomechanical parameters at 12 weeks: Mean value and standard deviation for specimens in the Low-Dose (LD) and High-Dose (HD) Groups.

Biomechanical Parameters at 12 weeks (Mean Value ± SD)	Low-Dose Group (n = 4)	High-Dose Group (n = 4)
Cross Sectional Area (mm^2^)	18.00 ± 7.45	10.29 ± 1.68
Stiffness (N/mm)	64.84 ± 5.9	61.02 ± 18.84
*Young’s* Modulus (MPa)	130.21 ± 50.19	233.55 ± 87.15
Yield Tension (N)	251.42 ± 100.80	205.23 ± 46.80
Yield Stress (MPa)	14.06 ± 0.72	20.01 ± 3.31
Ultimate Tension (N)	335.53 ± 83.78	247.99 ± 50.44
Ultimate Stress (MPa)	19.66 ± 3.58	24.58 ± 6.33

SD: standard deviation

n: number of animals

mm: millimeters

N: Newtons

MPa: Megapascal

**Table 5:** Biomechanical parameters at 16 weeks: Mean value and standard deviation (SD) for specimens in the Low-Dose (LD), High-Dose (HD) and control Groups.

Biomechanical Parameters at 16 weeks (Mean Value ± SD)	Control Group (n = 7)	Low-Dose Group (n = 5)	High-Dose Group (n = 4)
Cross Sectional Area (mm^2^)	9.58 ± 1.47	22.61 ± 14.35	11.74 ± 1.42
Stiffness (N/mm)	91.4 ± 23.7	60.338 ± 35.93	57.82 ± 16.06
*Young’s* Modulus (MPa)	300.1 ± 116.32	129.45 ± 81.76	191.18 ± 52.54
Yield Tension (N)	215.24 ± 55.88	155.34 ± 98.07	164.73 ± 62.67
Yield Stress (MPa)	23.45 ± 8.78	9.09 ± 6.72	14.47 ± 6.14
Ultimate Tension (N)	267.28 ± 50.11	213.84 ± 127.12	253.66 ± 95.32
Ultimate Stress (MPa)	28.86 ± 8.13	12.43 ± 8.93	22.39 ± 9.61

SD: standard deviation

n: number of animals

mm: millimeters

N: Newtons

MPa: Megapascal

After 10 weeks we found significant differences when comparing all biomechanical properties of the LD and HD groups, again with the exception of yield tension, with more pronounced findings and increased cross-sectional area in the HD group. After 12 weeks, the only significant difference found between the two groups was the cross-sectional area of the tendons, that was now increased in the specimens of the LD group. After 16 weeks, both experimental groups differed significantly from controls regarding decreased stiffness and yield stress. Only specimens of the LD group demonstrated significantly decreased Young’s modulus and ultimate stress, when compared to controls. The cross sectional area of the tendons were significantly increased in specimens of the LD group when compared to both HD group and controls. No differences were found between the cross-sectional area of specimens in the HD and control groups.

When comparing the different time points within the groups, we found significant differences in a few biomechanical properties of the LD group. After 16 weeks, the specimens demonstrated significantly decreased yield stress and ultimate stress when compared to the 10 week time-point, and decreased ultimate tension in relation to both 10 and 12 week time-points.

Specimens of the HD group demonstrated more pronounced findings, with increased cross sectional area of the tendon and decreased Young’s modulus, yield stress, and ultimate stress after 10 weeks, when compared to findings after 12 weeks. Significant differences were also found in the comparison between 10 and 16 week time-points, with the first demonstrating decreased yield stress and ultimate stress and increased cross-sectional area.

## Discussion

Tendinopathy is a common disease and can be debilitating. Despite the significant prevalence, its pathophysiology is not fully elucidated.[7] Animal models represent useful tools in the study of the physiopathology of the tendinopathy since human samples usually only come from late stage disease.[13,21,37–39] Bacterial collagenase type I, injected in a single large dose (0.3–0.5mg) is the most used technique in the literature for induction of tendinopathy in various animal models. However, to date, there were no studies evaluating the role of multiple consecutive injections of collagenase. We have shown in this study that serial application of a lower dose collagenase, when compared to a single high-dose injection, can induce a more progressive disease, with persistent findings after 16 weeks, replicating better in animals the histological and biomechanical findings proposed in the continuum model of human tendinopathy.[11]

In the histological analysis, after 10 weeks, tendinopathic findings were similarly pronounced in both HD and LD groups. Interestingly, for specimens of the HD group the pathological findings regressed after 12 weeks and remained diminished at the 16th week of follow-up. Some of these findings are similar to those reported in the literature for a single high-dose collagenase injection, with more intense tissue changes at 10 and 12 week follow-up, followed by partial healing and improvement of the Bonar score after 12 weeks.[22,40,41] However, differently from prior studies, there was no progression in the pathological process and chronification of the tendinopathy after 16th weeks.[16,22,27,32,42] The Bonar scores remained altered when compared to controls, but were similar to the scores observed at 12 weeks of follow-up.

Animals submitted to three low-dose injections of collagenase (LD group) demonstrated the same regression of the Bonar score at 12 weeks of follow-up, consistent with an attempt to heal. However, there was a progression of the pathological process after 16 weeks, with Bonar scores that were significantly higher than HD group and controls. Therefore, the histological findings for animals in LD group more accurately reproduced the progressive pathological process of tendinopathy as a continuum, with initial acute tendinopathic changes (10 weeks), followed by an attempt of healing (12 weeks) and further progression of the pathology (16 weeks), mimicking findings of human chronic tendinopathy.[11,43]

The biomechanical analysis demonstrated that after 10 weeks specimens of the HD group had the most pronounced changes. These include larger cross-sectional area, and lower stiffness, Young’s modulus, yield stress, ultimate stress and ultimate tension. These findings demonstrated that the application of the higher dose of collagenase led to more intense acute tissue changes. However, after 12 and 16 weeks the same findings were not present, what is consistent with the current literature, where tendons submitted to a single high-dose injection of collagenase displayed partial healing and possible recovery of biomechanical properties after 12 weeks.[32,44] The only biomechanical difference between the groups at 12th weeks was cross-sectional area of the tendons, that was significantly increased in the specimens of the LD group when compared to HD group.

After 16 weeks, the tendons in the LD group demonstrated a mean cross-sectional area approximately twice as large as HD group, and two and a half times the values from the controls. The values for HD group did not differ from the controls, which was different from the current data in the literature.[28,32,36] Significant differences were also observed after 16 weeks in the stiffness, Young’s modulus, yield stress, and ultimate stress when comparing specimens in the LD group and controls. For all these variables, LD group had significantly hindered biomechanical properties when compared to controls and a trend to more pronounced findings when compared to the HD group, that did not reach statistical significance. Our findings were similar to previous studies that evaluate these biomechanical parameters in tendons injected with a single high dose injection of collagenase.[17,25,28,29,32,36,44] However, this is the first time that the same tendency has been demonstrated for animals submitted to serial injections of lower dose collagenase. Although no significant differences were found when comparing LD and HD groups, it is important to consider that the relatively high loss rate of animals and the additional tissue sample exclusions affected the power of each sample.

Regarding comparison of biomechanical outcomes within each group (LD and HD) at different time points of euthanasia (10, 12 and 16 weeks), LD group demonstrated a trend towards a progressive intensification of alterations in the biomechanical properties of the tendons. However, statistical differences were found only for yield stress, ultimate stess and ultimate tension. In the HD group we found that several biomechanical properties (including cross sectional area, *Young’s* modulus, yield and ultimate stress) were significantly hindered after 10 weeks, demonstrating the more acute and destructive initial effect of a single high-dose injection of collagenase. Those alterations were however temporary and were not maintained after 12 and 16 weeks, again demonstrating the acute result of the single high-dose injection, with no chronification of the biomechanical findings.

We acknowledge several limitations of our study. We had a limited sample size that barely achieved the necessary number of animals in each group as calculated by our power analysis, what may have influenced the results of our study. The selection for histological analysis and scoring of tendinopathic findings was not completely random as noted in our methods, though it was taken from the same general area in all specimens and strictly followed standard analysis previously reported in the literature. [35] Also, animals in different groups underwent injections that contained different volumes of the solution. However, we have chosen to use the same concentration of the collagenase solution (10mg/ml) rather than the same volume. It is our understanding that using standardized concentration of collagenase injections would make more sense than using same volume of solution with different concentrations. We do not believe that injection of different volumes (10 or 30 microliters) would have altered the results significantly.

We also had significant losses of animals. We believe that is related mainly to anesthetic complications and the fact that rabbits represent a very fragile specimen, prone to perioperative complications. [38]

It is also important to acknowledge that some of the histological and biomechanical findings in the LD group could be related to an “acute” response to the last and third injection performed in animals of that group. However, at the time-point of 16 weeks, animals in that group had 10 weeks without any additional chemical aggression, making the demonstrated findings unlikely to be explained solely by the effects of the last injection.

## Conclusions

In conclusion, the biomechanical and histological findings in our study demonstrate that the serial application of lower doses (0.1mg) of collagenase type 1A in the Achilles tendon of rabbits results in a more progressive tendinopathic change when compared to the single higher dose (0.3mg) injection and to controls. The single collagenase injection model of induced tendinopathy, the most commonly used in the recent literature, might not be adequate enough to demonstrate the progressive development of the disease. We believe the proposed model of tendinopathy induced by serial injections of low-dose collagenase may represent a better option for the study of Achilles tendinopathy, with findings that mimic better the progressive nature of the chronic disease in humans. It is our hope that the results of this study can foster development of additional animal models of chronic Achilles tendinopathy, aiming more accurate elucidation of the pathophysiology of the disease and the search for better therapeutic possibilities.

## Supporting information

S1 Table Study raw data. Rabbit number, *Bonar* score reading for observer 1, *Bonar* score reading for observer 2, group (Low-Dose, High-Dose or Control), number of weeks for euthanasia, biomechanical parameters (cross sectional area, stiffness, *Young’s* modulus, yield tension, yield stress, ultimate tension and ultimate stress).
(XLSX)

S2 Table Complete statistical analysis. (1) Stiffness, (2) *Young’s* modulus, (3) yield tension, (4) yield stress, (5) ultimate tension, (6) ultimate stress, (7) cross sectional area, (8) histological *Bonar* score.
(DOCX)
