# Supplementary material for: Novel animal model for Achilles tendinopathy: Controlled experimental study of serial injections of collagenase in rabbits
Source: PLoS One. 2018 Feb 13;13(2):e0192769. doi: 10.1371/journal.pone.0192769 (PMC5811024; doi:10.1371/journal.pone.0192769)
Supplement: S2 Table — (1) Stiffness, (2) Young’s modulus, (3) yield tension, (4) yield stress, (5) ultimate tension, (6) ultimate stress, (7) cross sectional area, (8) histological Bonar score. (DOCX) [file pone.0192769.s002.docx]

**COMPLETE STATISTICAL ANALYSIS**

1. **Stiffness**

| **Group** | **Week** | **n** | **Mean** | **Std Dev** | **Minimum** | **Median** | **Maximum** |
| --- | --- | --- | --- | --- | --- | --- | --- |
| Control | 16 weeks | 7 | 91.4 | 23.71 | 43.67 | 92.01 | 113.91 |
| High-Dose | 10 weeks | 5 | 43.43 | 13.61 | 29.24 | 41.87 | 62.42 |
|  | 12 weeks | 4 | 61.02 | 18.84 | 33.06 | 69.21 | 72.61 |
|  | 16 weeks | 4 | 57.83 | 16.06 | 37.36 | 58.7 | 76.54 |
| Low-Dose | 10 weeks | 5 | 84.27 | 33.11 | 54.41 | 67.5 | 131.17 |
|  | 12 weeks | 4 | 64.85 | 5.9 | 59 | 64.25 | 71.89 |
|  | 16 weeks | 5 | 60.34 | 35.94 | 24.46 | 63.71 | 115.72 |

| **Source** | | **DF** | **Sum of Squares** | **Mean Square** | **F Value** | **p-value** |  |
| --- | --- | --- | --- | --- | --- | --- | --- |
| **Model** |  | 6 | 9115.522 | 1519.254 | 2.63 | 0.0388 |  |
|  | **Group** | 2 | 6717.966 | 3358.983 | 5.81 | 0.008 | * |
|  | **Week** | 2 | 164.023 | 82.011 | 0.14 | 0.8684 |  |
|  | **Group*Week** | 2 | 2233.534 | 1116.767 | 1.93 | 0.1644 |  |
| **Error** |  | 27 | 15609.920 | 578.145 |  |  |  |
| **Total** | | 33 | 24725.443 |  |  |  |  |

| **Week** | **Comparison between groups** | | | **Difference** | **CI (95%)** | |  |
| --- | --- | --- | --- | --- | --- | --- | --- |
| 10 weeks | High-Dose | - | Control | -47.97 | -76.85 | -19.08 | * |
|  | Low-Dose | - | Control | -7.13 | -36.02 | 21.76 |  |
|  | Low-Dose | - | High-Dose | 40.83 | 9.63 | 72.04 | * |
| 12 weeks | High-Dose | - | Control | -30.38 | -61.30 | 0.55 |  |
|  | Low-Dose | - | Control | -26.55 | -57.48 | 4.37 |  |
|  | Low-Dose | - | High-Dose | 3.83 | -31.06 | 38.71 |  |
| 16 weeks | High-Dose | - | Control | -33.58 | -64.50 | -2.65 | * |
|  | Low-Dose | - | Control | -31.06 | -59.95 | -2.17 | * |
|  | Low-Dose | - | High-Dose | 2.51 | -30.58 | 35.61 |  |

| **Group** | **Comparison between weeks** | | | **Difference** | **CI (95%)** | |
| --- | --- | --- | --- | --- | --- | --- |
| Low-Dose | 12 weeks | - | 10 weeks | -19.42 | -52.52 | 13.67 |
|  | 16 weeks | - | 10 weeks | -23.93 | -55.13 | 7.27 |
|  | 16 weeks | - | 12 weeks | -4.51 | -37.60 | 28.59 |
| High-Dose | 12 weeks | - | 10 weeks | 17.59 | -15.51 | 50.68 |
|  | 16 weeks | - | 10 weeks | 14.39 | -18.70 | 47.49 |
|  | 16 weeks | - | 12 weeks | -3.20 | -38.08 | 31.69 |

1. **Young's Modulus**

| **Group** | **Week** | **n** | **Mean** | **Std Dev** | **Minimum** | **Median** | **Maximum** |
| --- | --- | --- | --- | --- | --- | --- | --- |
| Control | 16 weeks | 7 | 301 | 116.32 | 134.84 | 286.92 | 512.78 |
| High-Dose | 10 weeks | 5 | 75 | 43.98 | 31.25 | 57.02 | 144.83 |
|  | 12 weeks | 4 | 233.56 | 87.15 | 113.07 | 251.6 | 317.95 |
|  | 16 weeks | 4 | 191.19 | 52.54 | 115.77 | 207.22 | 234.54 |
| Low-Dose | 10 weeks | 5 | 219.63 | 135.5 | 115.28 | 177.07 | 456.99 |
|  | 12 weeks | 4 | 130.22 | 50.17 | 73.03 | 135.12 | 177.59 |
|  | 16 weeks | 5 | 129.46 | 81.76 | 23.73 | 135.34 | 250.23 |

| **Source** | | **DF** | **Sum of Squares** | **Mean Square** | **F Value** | **p-value** |  |
| --- | --- | --- | --- | --- | --- | --- | --- |
| **Model** |  | 6 | 196972.836 | 32828.806 | 3.89 | 0.0063 |  |
|  | **Group** | 2 | 109372.805 | 54686.403 | 6.48 | 0.005 | * |
|  | **Week** | 2 | 5522.438 | 2761.219 | 0.33 | 0.7236 |  |
|  | **Group*Week** | 2 | 82077.594 | 41038.797 | 4.87 | 0.0157 | * |
| **Error** |  | 27 | 227727.953 | 8434.369 |  |  |  |
| **Total** | | 33 | 424700.789 |  |  |  |  |

| **Week** | **Comparison between groups** | | | **Difference** | **CI (95%)** | |  |
| --- | --- | --- | --- | --- | --- | --- | --- |
| 10 weeks | High-Dose | - | Control | -226.00 | -336.34 | -115.66 | * |
|  | Low-Dose | - | Control | -81.36 | -191.70 | 28.97 |  |
|  | Low-Dose | - | High-Dose | 144.64 | 25.46 | 263.81 | * |
| 12 weeks | High-Dose | - | Control | -67.44 | -185.55 | 50.67 |  |
|  | Low-Dose | - | Control | -170.78 | -288.89 | -52.67 | * |
|  | Low-Dose | - | High-Dose | -103.34 | -236.59 | 29.91 |  |
| 16 weeks | High-Dose | - | Control | -109.81 | -227.92 | 8.30 |  |
|  | Low-Dose | - | Control | -171.54 | -281.88 | -61.20 | * |
|  | Low-Dose | - | High-Dose | -61.73 | -188.13 | 64.68 |  |

| **Group** | **Comparison between weeks** | | | **Difference** | **CI (95%)** | |  |
| --- | --- | --- | --- | --- | --- | --- | --- |
| Low-Dose | 12 weeks | - | 10 weeks | -89.42 | -215.82 | 36.99 |  |
|  | 16 weeks | - | 10 weeks | -90.17 | -209.35 | 29.00 |  |
|  | 16 weeks | - | 12 weeks | -0.76 | -127.16 | 125.65 |  |
| High-Dose | 12 weeks | - | 10 weeks | 158.56 | 32.15 | 284.97 | * |
|  | 16 weeks | - | 10 weeks | 116.19 | -10.22 | 242.60 |  |
|  | 16 weeks | - | 12 weeks | -42.37 | -175.62 | 90.88 |  |

1. **Yield Tension**

| **Group** | **Week** | **n** | **Mean** | **Std Dev** | **Minimum** | **Median** | **Maximum** |
| --- | --- | --- | --- | --- | --- | --- | --- |
| Control | 16 weeks | 7 | 215.24 | 55.88 | 139.67 | 202.53 | 274.45 |
| High-Dose | 10 weeks | 5 | 117.56 | 42.37 | 68.79 | 102.79 | 180.02 |
|  | 12 weeks | 4 | 205.23 | 46.8 | 165.73 | 191.14 | 272.92 |
|  | 16 weeks | 4 | 164.73 | 62.68 | 77.45 | 183.77 | 213.93 |
| Low-Dose | 10 weeks | 5 | 271.1 | 87.4 | 138.01 | 268.12 | 366.81 |
|  | 12 weeks | 4 | 251.42 | 100.81 | 157.43 | 231.81 | 384.64 |
|  | 16 weeks | 5 | 155.34 | 98.08 | 65.84 | 109.25 | 283.19 |

| **Source** | | **DF** | **Sum of Squares** | **Mean Square** | **F Value** | **p-value** |
| --- | --- | --- | --- | --- | --- | --- |
| **Model** |  | 6 | 86293.182 | 14382.197 | 2.7 | 0.0348 |
|  | **Group** | 2 | 31356.788 | 15678.394 | 2.94 | 0.0697 |
|  | **Week** | 2 | 22383.277 | 11191.639 | 2.1 | 0.1418 |
|  | **Group*Week** | 2 | 32553.116 | 16276.558 | 3.06 | 0.0636 |
| **Error** |  | 27 | 143790.319 | 5325.567 |  |  |
| **Total** | | 33 | 230083.501 |  |  |  |

1. **Yield Stress**

| **Group** | **Week** | **n** | **Mean** | **Std Dev** | **Minimum** | **Median** | **Maximum** |
| --- | --- | --- | --- | --- | --- | --- | --- |
| Control | 16 weeks | 7 | 23.46 | 8.78 | 11.81 | 22.42 | 37.51 |
| High-Dose | 10 weeks | 5 | 4.73 | 2.15 | 2.11 | 6.01 | 6.71 |
|  | 12 weeks | 4 | 20.01 | 3.31 | 15.73 | 20.31 | 23.7 |
|  | 16 weeks | 4 | 14.47 | 6.14 | 5.71 | 16.13 | 19.93 |
| Low-Dose | 10 weeks | 5 | 17.91 | 9.93 | 9.51 | 14.02 | 34.82 |
|  | 12 weeks | 4 | 14.06 | 0.73 | 13.09 | 14.16 | 14.85 |
|  | 16 weeks | 5 | 9.09 | 6.72 | 1.38 | 6.27 | 16.4 |

| **Source** | | **DF** | **Sum of Squares** | **Mean Square** | **F Value** | **p-value** |  |
| --- | --- | --- | --- | --- | --- | --- | --- |
| **Model** |  | 6 | 1348.812 | 224.802 | 5.04 | 0.0014 |  |
|  | **Group** | 2 | 610.096 | 305.048 | 6.84 | 0.004 | * |
|  | **Week** | 2 | 181.362 | 90.681 | 2.03 | 0.1504 |  |
|  | **Group*Week** | 2 | 557.355 | 278.677 | 6.25 | 0.0059 | * |
| **Error** |  | 27 | 1204.036 | 44.594 |  |  |  |
| **Total** | | 33 | 2552.848 |  |  |  |  |

| **Week** | **Comparison between groups** | | | **Difference** | **CI (95%)** | |  |
| --- | --- | --- | --- | --- | --- | --- | --- |
| 10 weeks | High-Dose | - | Control | -18.73 | -26.75 | -10.71 | * |
|  | Low-Dose | - | Control | -5.55 | -13.57 | 2.48 |  |
|  | Low-Dose | - | High-Dose | 13.18 | 4.52 | 21.85 | * |
| 12 weeks | High-Dose | - | Control | -3.44 | -12.03 | 5.14 |  |
|  | Low-Dose | - | Control | -9.39 | -17.98 | -0.81 | * |
|  | Low-Dose | - | High-Dose | -5.95 | -15.64 | 3.74 |  |
| 16 weeks | High-Dose | - | Control | -8.98 | -17.57 | -0.40 | * |
|  | Low-Dose | - | Control | -14.36 | -22.39 | -6.34 | * |
|  | Low-Dose | - | High-Dose | -5.38 | -14.57 | 3.81 |  |

| **Group** | **Comparison between weeks** | | | **Difference** | **CI (95%)** | |  |
| --- | --- | --- | --- | --- | --- | --- | --- |
| Low-Dose | 12 weeks | - | 10 weeks | -3.85 | -13.04 | 5.34 |  |
|  | 16 weeks | - | 10 weeks | -8.82 | -17.48 | -0.15 | * |
|  | 16 weeks | - | 12 weeks | -4.97 | -14.16 | 4.22 |  |
| High-Dose | 12 weeks | - | 10 weeks | 15.29 | 6.10 | 24.48 | * |
|  | 16 weeks | - | 10 weeks | 9.75 | 0.56 | 18.94 | * |
|  | 16 weeks | - | 12 weeks | -5.54 | -15.23 | 4.15 |  |

1. **Ultimate Tension**

| **Group** | **Week** | **n** | **Mean** | **Std Dev** | **Minimum** | **Median** | **Maximum** |
| --- | --- | --- | --- | --- | --- | --- | --- |
| Control | 16 weeks | 7 | 267.29 | 50.12 | 163.03 | 275.63 | 311.85 |
| High-Dose | 10 weeks | 5 | 168.17 | 59.6 | 100.62 | 202.42 | 216.25 |
|  | 12 weeks | 4 | 248 | 50.45 | 203.53 | 240.74 | 306.99 |
|  | 16 weeks | 4 | 253.67 | 95.32 | 110.82 | 299.46 | 304.94 |
| Low-Dose | 10 weeks | 5 | 389.14 | 105.68 | 222.28 | 388.75 | 503.38 |
|  | 12 weeks | 4 | 335.53 | 83.78 | 260.72 | 333.16 | 415.1 |
|  | 16 weeks | 5 | 213.84 | 127.13 | 111.49 | 129.06 | 384.93 |

| **Source** | | **DF** | **Sum of Squares** | **Mean Square** | **F Value** | **p-value** |  |
| --- | --- | --- | --- | --- | --- | --- | --- |
| **Model** |  | 6 | 158503.208 | 26417.201 | 3.67 | 0.0086 |  |
|  | **Group** | 2 | 57275.577 | 28637.789 | 3.97 | 0.0307 | * |
|  | **Week** | 2 | 21047.272 | 10523.636 | 1.46 | 0.2499 |  |
|  | **Group*Week** | 2 | 80180.359 | 40090.179 | 5.56 | 0.0095 | * |
| **Error** |  | 27 | 194549.179 | 7205.525 |  |  |  |
| **Total** | | 33 | 353052.388 |  |  |  |  |

| **Week** | **Comparison between groups** | | | **Difference** | **CI (95%)** | |  |
| --- | --- | --- | --- | --- | --- | --- | --- |
| 10 weeks | High-Dose | - | Control | -99.12 | -201.10 | 2.87 |  |
|  | Low-Dose | - | Control | 121.86 | 19.87 | 223.84 | * |
|  | Low-Dose | - | High-Dose | 220.97 | 110.82 | 331.13 | * |
| 12 weeks | High-Dose | - | Control | -19.29 | -128.46 | 89.88 |  |
|  | Low-Dose | - | Control | 68.24 | -40.92 | 177.41 |  |
|  | Low-Dose | - | High-Dose | 87.54 | -35.62 | 210.69 |  |
| 16 weeks | High-Dose | - | Control | -13.62 | -122.79 | 95.55 |  |
|  | Low-Dose | - | Control | -53.45 | -155.43 | 48.54 |  |
|  | Low-Dose | - | High-Dose | -39.83 | -156.66 | 77.01 |  |

| **Group** | **Comparison between weeks** | | | **Difference** | **CI (95%)** | |  |
| --- | --- | --- | --- | --- | --- | --- | --- |
| Low-Dose | 12 weeks | - | 10 weeks | -53.61 | -170.45 | 63.23 |  |
|  | 16 weeks | - | 10 weeks | -175.30 | -285.46 | -65.15 | * |
|  | 16 weeks | - | 12 weeks | -121.69 | -238.53 | -4.86 | * |
| High-Dose | 12 weeks | - | 10 weeks | 79.83 | -37.01 | 196.66 |  |
|  | 16 weeks | - | 10 weeks | 85.50 | -31.34 | 202.33 |  |
|  | 16 weeks | - | 12 weeks | 5.67 | -117.49 | 128.83 |  |

1. **Ultimate Stress**

| **Group** | **Week** | **n** | **Mean** | **Std Dev** | **Minimum** | **Median** | **Maximum** |
| --- | --- | --- | --- | --- | --- | --- | --- |
| Control | 16 weeks | 7 | 28.86 | 8.13 | 13.79 | 29.84 | 41.3 |
| High-Dose | 10 weeks | 5 | 7.13 | 4.16 | 2.87 | 8.04 | 12.64 |
|  | 12 weeks | 4 | 24.59 | 6.33 | 17.53 | 23.94 | 32.93 |
|  | 16 weeks | 4 | 22.4 | 9.61 | 8.18 | 26.25 | 28.9 |
| Low-Dose | 10 weeks | 5 | 25.08 | 10.59 | 15.32 | 21.09 | 42.73 |
|  | 12 weeks | 4 | 19.67 | 3.58 | 15.23 | 19.74 | 23.96 |
|  | 16 weeks | 5 | 12.43 | 8.93 | 2.33 | 9.88 | 21.87 |

| **Source** | | **DF** | **Sum of Squares** | **Mean Square** | **F Value** | **p-value** |  |
| --- | --- | --- | --- | --- | --- | --- | --- |
| **Model** |  | 6 | 1896.612 | 316.102 | 5.11 | 0.0013 |  |
|  | **Group** | 2 | 661.208 | 330.604 | 5.35 | 0.0111 | * |
|  | **Week** | 2 | 185.604 | 92.802 | 1.5 | 0.241 |  |
|  | **Group*Week** | 2 | 1049.801 | 524.901 | 8.49 | 0.0014 | * |
| **Error** |  | 27 | 1669.662 | 61.839 |  |  |  |
| **Total** | | 33 | 3566.274 |  |  |  |  |

| **Week** | **Comparison between groups** | | | **Difference** | **CI (95%)** | |  |
| --- | --- | --- | --- | --- | --- | --- | --- |
| 10 weeks | High-Dose | - | Control | -21.73 | -31.18 | -12.28 | * |
|  | Low-Dose | - | Control | -3.78 | -13.23 | 5.67 |  |
|  | Low-Dose | - | High-Dose | 17.95 | 7.75 | 28.15 | * |
| 12 weeks | High-Dose | - | Control | -4.28 | -14.39 | 5.84 |  |
|  | Low-Dose | - | Control | -9.20 | -19.31 | 0.92 |  |
|  | Low-Dose | - | High-Dose | -4.92 | -16.33 | 6.49 |  |
| 16 weeks | High-Dose | - | Control | -6.47 | -16.58 | 3.65 |  |
|  | Low-Dose | - | Control | -16.43 | -25.88 | -6.99 | * |
|  | Low-Dose | - | High-Dose | -9.97 | -20.79 | 0.86 |  |

| **Group** | **Comparison between weeks** | | | **Difference** | **CI (95%)** | |  |
| --- | --- | --- | --- | --- | --- | --- | --- |
| Low-Dose | 12 weeks | - | 10 weeks | -5.42 | -16.24 | 5.41 |  |
|  | 16 weeks | - | 10 weeks | -12.65 | -22.86 | -2.45 | * |
|  | 16 weeks | - | 12 weeks | -7.24 | -18.06 | 3.59 |  |
| High-Dose | 12 weeks | - | 10 weeks | 17.45 | 6.63 | 28.27 | * |
|  | 16 weeks | - | 10 weeks | 15.26 | 4.44 | 26.08 | * |
|  | 16 weeks | - | 12 weeks | -2.19 | -13.60 | 9.22 |  |

1. **Cross-Sectional Area**

| **Group** | **Week** | **n** | **Mean** | **Std Dev** | **Minimum** | **Median** | **Maximum** |
| --- | --- | --- | --- | --- | --- | --- | --- |
| Control | 16 weeks | 7 | 9.59 | 1.48 | 6.98 | 9.89 | 11.82 |
| High-Dose | 10 weeks | 5 | 27.17 | 7.88 | 17.11 | 26.84 | 36.87 |
|  | 12 weeks | 4 | 10.29 | 1.69 | 8.42 | 10.42 | 11.9 |
|  | 16 weeks | 4 | 11.75 | 1.42 | 10.5 | 11.47 | 13.55 |
| Low-Dose | 10 weeks | 5 | 16.6 | 5.4 | 10.53 | 14.87 | 24.99 |
|  | 12 weeks | 4 | 18.01 | 7.45 | 11.09 | 16.84 | 27.26 |
|  | 16 weeks | 5 | 22.61 | 14.36 | 13.06 | 17.77 | 47.83 |

| **Group** | **Group2** | **n** | **Mean** | **Std Dev** | **Minimum** | **Median** | **Maximum** |
| --- | --- | --- | --- | --- | --- | --- | --- |
| Control | 16 weeks | 7 | 2.25 | 0.16 | 1.94 | 2.29 | 2.47 |
| High-Dose | 10 weeks | 5 | 3.27 | 0.3 | 2.84 | 3.29 | 3.61 |
|  | 12 weeks | 4 | 2.32 | 0.17 | 2.13 | 2.34 | 2.48 |
|  | 16 weeks | 4 | 2.46 | 0.12 | 2.35 | 2.44 | 2.61 |
| Low-Dose | 10 weeks | 5 | 2.77 | 0.32 | 2.35 | 2.7 | 3.22 |
|  | 12 weeks | 4 | 2.83 | 0.42 | 2.41 | 2.8 | 3.31 |
|  | 16 weeks | 5 | 3 | 0.52 | 2.57 | 2.88 | 3.87 |

| **Source** | | **DF** | **Sum of Squares** | **Mean Square** | **F Value** | **p-value** |  |
| --- | --- | --- | --- | --- | --- | --- | --- |
| **Model** |  | 6 | 4.349 | 0.725 | 7.41 | <.0001 |  |
|  | **Group** | 2 | 1.809 | 0.904 | 9.24 | 0.0009 | * |
|  | **Week** | 2 | 0.906 | 0.453 | 4.63 | 0.0187 | * |
|  | **Group*Week** | 2 | 1.635 | 0.817 | 8.36 | 0.0015 | * |
| **Error** |  | 27 | 2.641 | 0.098 |  |  |  |
| **Total** | | 33 | 6.991 |  |  |  |  |

| **Week** | **Comparison between groups** | | | **Difference** | **CI (95%)** | |  |
| --- | --- | --- | --- | --- | --- | --- | --- |
| 10 weeks | High-Dose | - | Control | 1.02 | 0.64 | 1.39 | * |
|  | Low-Dose | - | Control | 0.52 | 0.14 | 0.89 | * |
|  | Low-Dose | - | High-Dose | -0.50 | -0.90 | -0.09 | * |
| 12 weeks | High-Dose | - | Control | 0.07 | -0.33 | 0.47 |  |
|  | Low-Dose | - | Control | 0.58 | 0.17 | 0.98 | * |
|  | Low-Dose | - | High-Dose | 0.50 | 0.05 | 0.96 | * |
| 16 weeks | High-Dose | - | Control | 0.21 | -0.19 | 0.61 |  |
|  | Low-Dose | - | Control | 0.75 | 0.37 | 1.12 | * |
|  | Low-Dose | - | High-Dose | 0.54 | 0.11 | 0.97 | * |

| **Group** | **Comparison between weeks** | | | **Difference** | **CI (95%)** | |  |
| --- | --- | --- | --- | --- | --- | --- | --- |
| Low-Dose | 12 weeks | - | 10 weeks | 0.06 | -0.37 | 0.49 |  |
|  | 16 weeks | - | 10 weeks | 0.23 | -0.18 | 0.63 |  |
|  | 16 weeks | - | 12 weeks | 0.17 | -0.26 | 0.60 |  |
| High-Dose | 12 weeks | - | 10 weeks | -0.95 | -1.38 | -0.51 | * |
|  | 16 weeks | - | 10 weeks | -0.81 | -1.24 | -0.38 | * |
|  | 16 weeks | - | 12 weeks | 0.14 | -0.32 | 0.59 |  |

1. **Histological Bonar Score**

| **Group** | **Time-Point** | **n** | **Mean** | **Std Dev** | **Minimum** | **Median** | **Maximum** |
| --- | --- | --- | --- | --- | --- | --- | --- |
| Control | 16 weeks | 8 | 2 | 0.82 | 1 | 2 | 3 |
| High-Dose | 10 weeks | 5 | 12.8 | 0.84 | 12 | 13 | 14 |
|  | 12 weeks | 4 | 4.5 | 1.91 | 2 | 5 | 6 |
|  | 16 weeks | 5 | 5.6 | 2.51 | 2 | 5 | 8 |
| Low-Dose | 10 weeks | 5 | 11.6 | 0.55 | 11 | 12 | 12 |
|  | 12 weeks | 6 | 8 | 2.1 | 6 | 7.5 | 11 |
|  | 16 weeks | 5 | 11.8 | 2.28 | 8 | 12 | 14 |

| **Source** | | **DF** | **Sum of Squares** | **Mean Square** | **F Value** | **p-value** |  |
| --- | --- | --- | --- | --- | --- | --- | --- |
| **Model** |  | 6 | 580.676 | 96.779 | 33.37 | <.0001 |  |
|  | **Group** | 2 | 336.524 | 168.262 | 58.02 | <.0001 | * |
|  | **Week** | 2 | 174.080 | 87.040 | 30.01 | <.0001 | * |
|  | **Group*Week** | 2 | 70.072 | 35.036 | 12.08 | 0.0001 | * |
| **Error** |  | 30 | 87.000 | 2.900 |  |  |  |
| **Total** | | 36 | 667.676 |  |  |  |  |

| **Week** | **Comparison between groups** | | | **Difference** | **CI (95%)** | |  |
| --- | --- | --- | --- | --- | --- | --- | --- |
| 10 weeks | High-Dose | - | Control | 10.80 | 8.76 | 12.84 | * |
|  | Low-Dose | - | Control | 9.60 | 7.56 | 11.64 | * |
|  | Low-Dose | - | High-Dose | -1.20 | -3.40 | 1.00 |  |
| 12 weeks | High-Dose | - | Control | 2.50 | 0.32 | 4.68 | * |
|  | Low-Dose | - | Control | 6.00 | 4.07 | 7.93 | * |
|  | Low-Dose | - | High-Dose | 3.50 | 1.26 | 5.74 | * |
| 16 weeks | High-Dose | - | Control | 3.60 | 1.56 | 5.64 | * |
|  | Low-Dose | - | Control | 9.80 | 7.76 | 11.84 | * |
|  | Low-Dose | - | High-Dose | 6.20 | 4.00 | 8.40 | * |

| **Group** | **Comparison between weeks** | | | **Difference** | **CI (95%)** | |  |
| --- | --- | --- | --- | --- | --- | --- | --- |
| Low-Dose | 12 weeks | - | 10 weeks | -3.60 | -5.71 | -1.49 | * |
|  | 16 weeks | - | 10 weeks | 0.20 | -2.00 | 2.40 |  |
|  | 16 weeks | - | 12 weeks | 3.80 | 1.69 | 5.91 | * |
| High-Dose | 12 weeks | - | 10 weeks | -8.30 | -10.63 | -5.97 | * |
|  | 16 weeks | - | 10 weeks | -7.20 | -9.40 | -5.00 | * |
|  | 16 weeks | - | 12 weeks | 1.10 | -1.23 | 3.43 |  |
